# The digital transformation of surgery

**DOI:** 10.1038/s41746-023-00846-3

**Published:** 2023-05-31

**Authors:** Jayson S. Marwaha, Marium M. Raza, Joseph C. Kvedar

**Affiliations:** 1 Beth Israel Deaconess Medical Center, Boston, MA USA; 2 Harvard Medical School, Boston, MA USA; 3 Mass General Brigham, Boston, MA USA

**Keywords:** Translational research, Health services

## Abstract

Rapid advances in digital technology and artificial intelligence in recent years have already begun to transform many industries, and are beginning to make headway into healthcare. There is tremendous potential for new digital technologies to improve the care of surgical patients. In this piece, we highlight work being done to advance surgical care using machine learning, computer vision, wearable devices, remote patient monitoring, and virtual and augmented reality. We describe ways these technologies can be used to improve the practice of surgery, and discuss opportunities and challenges to their widespread adoption and use in operating rooms and at the bedside.

Innovations in digital technology have begun to transform the practice of medicine. From FDA approved artificial intelligence systems for endoscopy^1^ to the growing use of wearable biosensors in clinical trials^2^, digital technologies are already being applied within various fields of medicine. Historically the field of surgery has justifiably been relatively cautious with deployment of new and potentially disruptive technologies that have not been extensively studied, given the possibility of direct and immediate patient harm^3^; surgery’s experience with digital technologies so far is no different. However, with the tremendous potential for new digital technologies to improve surgical care, we believe its incorporation into daily surgical practice is inevitable. Indeed fields such as gastroenterology and radiology are already incorporating digital technologies in their practice^4,5^. In this npj Digital Medicine special collection, we highlight work being done in several areas of digital technology—machine learning, computer vision, wearable devices, remote patient monitoring, and virtual and augmented reality—all of which we believe are poised to transform surgery in the coming years. In this piece, we describe opportunities and challenges to these technologies’ adoption.

## Emerging technologies

### Machine learning-enabled clinical decision support

Machine learning models have the potential to learn complex, nuanced relationships between enormous numbers of clinical variables, including multimodal data, in ways that traditional statistical risk calculators cannot. This advantage may soon be leveraged to better predict the clinical trajectory of patients and help surgeons make more personalized patient care decisions.

Specifically, AI enabled decision support systems have the potential to predict surgical outcomes. Surgical outcome prediction is important for patient care both preoperatively, in deciding which patients would be candidates for benefit with surgical intervention, as well as postoperatively, in predicting risk of complications. Machine learning algorithms can be applied to both aspects to assist outcome prediction. Mobile applications with these kinds of algorithms have been developed and piloted at multiple large academic institutions^6–9^.

Highlighted in this collection, a review of machine learning models that learned from clinical data in vascular surgery found that several models perform better than existing clinical prediction tools, clinicians, and traditional regression models^10^. The study further finds that as machine learning tools are increasingly applied to vascular surgery, model performance continues to improve with newer studies. These findings emphasize that machine learning may one day serve as an important augmentation for analysis, diagnosis, and outcome prediction in surgery.

### Computer vision and augmented reality

Computer vision—the application of machine learning algorithms for visual data analysis—holds tremendous potential to impact clinical care wherever images or video data are involved. In this special collection we focus mainly on applications of computer vision for analysis of intraoperative data. Rapid advancements in the field of computer vision, including the increasing use of deep neural networks, have led to the development of algorithms that can accurately identify clinically important aspects of intraoperative video data^11^. Among the most exciting potential applications of real-time intraoperative computer vision analysis to support safer surgery is providing an augmented reality experience for surgeons to aid in intraoperative decision-making^12^. Many existing published computer vision models have demonstrated the ability to assess operative complexity, assist with decision-making during minimally invasive procedures, assess the technical skill of surgeons in an automated and scalable fashion, provide intraoperative feedback, assess OR team dynamics, and even predict postoperative outcomes based on intraoperative events^13–15^. When combined with these technologies, virtual reality can be used as a training ground for operative skills assessment and education^16^. While the incorporation of these tools in regular surgical practice is not yet a reality, potential use cases will surely continue to grow as research in this field continues.

### Wearable devices and remote patient monitoring

Wearable devices and other personal technologies that collect and transmit physiologic signals and patient reported variables in real time are another important technology with the potential to improve surgery. The use of wearable devices for remote patient monitoring, which enables evaluation of patients beyond traditional in-person encounters with the healthcare system, holds great opportunity for the improvement of postoperative care by enabling remote assessment of surgical wounds, functional status, pain control, and detecting clinical deterioration early. There is emerging evidence that this form of data capture can potentially reduce complication rates^17^. In our collection, Mori et al. use a digital health platform to better understand the nuances of post operative recovery in the home for cardiac surgery patients, far beyond just clinical events captured during postoperative ED visits or clinic visits^18^.

In addition to understanding the postoperative course overall, recent work shows the potential for remote patient monitoring changing the way postoperative care is delivered. A randomized control trial finds that postoperative surveillance of surgical site infection using a smartphone is just as effective as in person follow-up assessment^19^, suggesting that the use of wearable devices for remote patient wound monitoring may be safe, efficient, and reduce health service utilization. Another study shows that the use of a non-contact video camera to monitor vital signs can be used to track early signs of physiological deterioration, to function as a less disruptive, more efficient, early warning system^20^. These new capabilities may enable an eventual shift in the nucleus of post-surgical care from the hospital back to the home; some large academic medical centers have already established successful digital technology-enabled surgical home hospital programs that allow patients to be safely recover from surgery in their own home^21^.

### Cautions and considerations

While there are many emerging digital technologies that hold great promise for surgery, there are also important cautions and considerations for their use.

One important concern is equity. There are disparities in surgical outcomes for patients of racial minorities as well as lower socioeconomic status that persist until today. Just one example is in spine surgery, where the risk of postoperative complications has been estimated to be as much as 61% higher for Black patients^22^. Without a critical assessment of digital technologies from an equity lens, these disparities may continue to be perpetuated. Halamka et al. highlight the importance of using existing bias detection tools to detect irregularities in datasets and algorithms before deploying them in surgical practice^23^.

Another important concern is information security. Operating rooms are growing in terms of technological sophistication and the use of connected devices. Surgical robots for example, are now being widely used at growing rates around the world and increasingly rely on constant connectivity to deliver real-time insights and support. At the same time, the number of cyberattacks on hospitals has also been significantly increasing^24^. The rapid adoption of sophisticated connected technologies in operating rooms makes them uniquely susceptible to targeted attacks that can cause patient harm. An important contribution in this collection by Gordon et al. explains how health systems can mitigate the risks of cybersecurity attacks in operating rooms, including minimizing real-time connectivity during surgical procedures to only what is necessary, collaborating with vendors on developing safety measures, and establishing downtime procedures^25^.

A third important consideration is determining how these technologies will be regulated to ensure they are safe and effective. One unique element of AI-enabled technologies is their ability to adapt over time as they are introduced to new data. This may cause unanticipated shifts in performance and ultimately a very different tool from the one that was originally approved. The US Food and Drug Administration (FDA) is in the process of determining how to best regulate these tools. They developed a proposed regulatory framework in 2019^26^ and have since been refining it; most recently in April 2023 they put forth a new draft guidance that recommends continuous product monitoring throughout the product lifecycle with real-world data and allows for small adjustments in performance over time^27^.

Among the most important barriers to widespread adoption of these technologies in surgery is limited evidence of their clinical impact^28^. For example, few studies have shown any improvement in care when AI-enabled decision support tools are used^29^. Some important barriers to clinical impact include translating ML model outputs into clinically actionable insights and incorporating them into existing workflows and clinical decision making processes^15,21,30^. Additionally, these new technologies must be built and rigorously evaluated in a way that is reproducible, transparent, and interpretable to users to earn their trust^30,31^. Access to larger and more diverse datasets may help address some of these issues^12^.

### Looking ahead

The number of new digital technologies with the potential to improve surgical care is rapidly growing. There are many use cases and technologies not covered in this special collection. Generative AI models, such as GPT-4 and DALL-E 2, are only just beginning to be explored in the healthcare literature, yet their tremendous abilities have sparked widespread interest in exploring potential use cases^32,33^. As another example, the use of ambient sensing monitors in operating rooms may help automate quality control processes, prevent medical errors, and improve a surgical team’s overall perioperative situational awareness^34^.

Research in this field should focus on bridging the gap from in silico performance to real-world clinical benefit of new digital technologies. This will involve building out the implementation science of these tools, evaluating them with real-world evidence, and continuing to explore new applications^35^. Health systems will need to begin investing in the expertise and IT infrastructure necessary to deploy and maintain these tools^36^. The augmentation of surgery with these emerging digital technologies is necessary for progress toward more efficient and effective patient care.
